# The Relationship between Prostate Biopsy Results and PSA and Free PSA Ratio Changes in Elevated Serum PSA Patients with and without Antibiotherapy

**DOI:** 10.31557/APJCP.2020.21.4.1051

**Published:** 2020-04

**Authors:** Mesut Berkan Duran, Ayhan Dirim, Hakan Ozkardes

**Affiliations:** 1 *Samsun Training and Research Hospital, Department of Urology, Samsun, *; 2 *Department of Urology, Baskent University School of Medicine, Ankara, Turkey. *

**Keywords:** Benign prostatic hyperplasia, chronic prostatitis, empiric antibiotic treatment, prostate biopsy, prostate cancer

## Abstract

**Objectives:**

To evaluate the impact of antibiotic treatment on total prostate specific antigen (PSA) levels and free/total (f/t) PSA ratio and the relevance of these changes to prostate biopsy results.

**Methods:**

We retrospectively evaluated 1,062 patients with elevated age-adjusted serum PSA levels who underwent prostate biopsy between 2004 and 2016. A total of 303 cases with followup PSA levels and f/t PSA ratio before and after antibiotherapy were included into this study. There were 214 patients with persistent elevated serum PSA levels after antibiotic treatment followed by prostate biopsy (treatment group) and 89 patients who had prostate biopsy after a mean followup of 1 month without antibiotherapy (control group). The groups were compared with regard to both 5% and 10% cut off changes in serum PSA levels and f/t PSA ratios.

**Results:**

Antibiotic treatment had no impact on the relation between serum PSA levels and biopsy results at both cut off values. On the other hand, f/t PSA ratio changes at both cut off values with relevance to antibiotic treatment were found to be related with histopathologic results. While increase in f/t PSA ratio was more related with benign biopsies, decrease in f/t PSA ratio was more related with cancer (for 5% cut off value p= 0.014, p= 0.004; for 10% cut off value p= 0.026, p= 0.014).

**Conclusion:**

Changes at f/t PSA ratio rather than total PSA only, particularly in antibiotic treated cases appear to be more useful in decision making for biopsy.

## Introduction

Serum PSA levels may vary in healthy individuals based on age, race and prostatic volume. It may increase with trauma, ejaculation, transurethral interventions, or trans-rectal ultrasonography, may also increase with diseases such as benign prostate hyperplasia (BPH) or acute/chronic prostatitis other than prostate cancer. Increasing levels of serum PSA related with sub-clinic inflammation is one of the most common reasons for negative prostate biopsies that have been performed for the diagnosis of prostate cancer. Making the decision for prostate biopsy in cases with normal rectal digital examination and elevated age related PSA findings is one of the issues that lead the urologysts to a dead end. Eliminating prostatic inflammation with antibiotherapy to make the decision for biopsy, carrying out the biopsy directly or taking the changes in PSA levels and re-evaluating the PSA levels after a reasonable period of time before directing patients to biopsy are among the approaches used frequently. The issue of avoiding the unnecessary biopsies with the use of antibiotics and on the other hand, delaying the early diagnosis still maintains its currency among the topics dicussed the most. Multiparametric magnetic resonance imaging (mpMRI) has been increasingly used for guiding several aspects of prostate cancer management, including detection, staging, and treatment. The application of mpMRI for diagnosis of prostate cancer has been recommended by the European Association of Urology (EAU), National Comprehensive Cancer Network (NCCN), and European Society of Urogenital Radiology (ESUR) guidelines. However results of mpMRI may vary sharply because of difficulties in interpretation, lacking of standardized criteria for positive definition and the ability of radiologists (Kim et al., 2014). Besides, the disadvantages of mpMRI such as equipment-specialization, limitation of accessibility and time-consuming also impede its wide application (Sartor et al., 2008).

## Materials and Methods

One thousand and sixty-two patients who had underwent prostatic biopsy guided by TRUS in Başkent University Medical School, Urology Department between 2004 and 2016 were evaluated retrospectively. Cases with abnormal findings in the rectal examination (suspicion of malignancy such as asymmetry, nodule, induration) and those using drugs that can affect the PSA levels were excluded from the study. 303 patients with normal rectal examination and high age related PSA levels who had underwent prostate biopsy procedure with the accompaniment of TRUS (clinical stage T1c) were evaluated. All the patients were evaluated after dividing them into 4 sub-groups as the cases with total PSA value and f/t PSA ratio before and after the antibiotherapy in the treatment group and the cases with control PSA and control f/t PSA ratio in the control group. It was determined whether or not to give antibiotic treatment according to the physician’s preferences. Patients were checked with f/t PSA and tPSA after a while with or without antibiotic administration due to dysuria, leucocyturia or pain without urinary tract infection. Changes in total PSA and f/t PSA ratios in the treatment and control groups were evaluated based on 5% and 10% cut off values. In this study, it is aimed to evaluate the effects of antibiotherapy to serum PSA levels and f/t PSA ratio and its reflection to biopsy results. 

Study data were uploaded to the computer using the “SPSS (Statistical Package for Social Sciences) for Windows 22.0 (SPSS Inc, Chicago, IL)” and analyzed. Descriptive statistics were expressed as the median (minimum-maximum), frequency distribution and percentage. Pearson chi-square test, Fisher’s exact test and chi-square test for single sample were used for the evaluation of categorical variables. Conformance of the variables with normal distribution was examined using visual (based on histograms and probability curves) and analytical methods (Kolmogorov-Smirnov Test). As regards the variables found out as nonconforming with normal distribution, Mann-Whitney U Test was used as the statistical method for the statistical significance between two independent groups, and Wilcoxon’s signed rank test was used for two dependent groups. Statistical significance level was accepted as p <0.05.

## Results

The median age of the 303 cases included in the study was 63 years (45-86 years). While antibiotherapy had been used in 70.6% (n= 214) of patients (treatment group), it had not been used in 29.4% (n= 89) (control group). The median period for antibiotic use was 4 weeks (2-8 weeks), and ciprofloxacin was the most frequently used antibiotic (47.4%). The total PSA and the free/total PSA ratio changes in the treatment and control groups are summarized in Table 1. The increase in PSA values in the control group was found as significant (p <0.001). Also, significant decreases of f/t PSA was observed in the treatment group as compared to the control group (p= 0.027). 

When evaluated in terms of histopathological results; prostate biopsy was reported as cancer in 103 cases and benign in 200 cases. The changes in total PSA and f/t PSA ratio in the treatment and control groups for 5% cut-off value and prostate biopsy results are shown in Table 2. No correlation was found between the total PSA values and histopathological results in both groups. In the evaluation based on the changes in the free/total PSA ratio, the prostate cancer rate was found as significantly higher in patients with antibiotic use and decreasing f/t PSA ratio (p= 0.004). Again, benign pathologies were significantly higher in patients with increasing rates of f/t PSA ratio in treatment group (p= 0.014) (Table 2). 

For the cut-off value of 10% in treatment and control groups, the changes in total PSA and f/t PSA ratio and prostate biopsy results are shown in Table 3. No relationship was found between the changes in total PSA values and histopathological results for the cut-off value of 10%. In the evaluation based on changes in free/total PSA ratio, prostate cancer rate was found significantly higher in patients receiving antibiotics and with decreasing s/t PSA ratios (p= 0.014). Also, benign pathologies were found significantly higher in cases with increasing f/t PSA ratios (Table 3). 

Distribution of pathology results based on groups of change in f/t PSA ratio for cut-off values of 5% and 10%, 194 patients in total among the patients included to the study in the two groups in relation with f/t PSA values before and after are shown in Table 4. For the cut-off value of 5%, cancer was found significantly higher in cases with decreasing f/t PSA ratio, and benign pathologies were found significantly higher in cases with decreasing f/t PSA ratio (p= 0.005 and p= 0.014, respectively). For the cut-off value of 10%, only the percentage of cancer was significantly higher in cases that f/t PSA ratio had decreased (p= 0.025) (Table 4).

**Table 1.** Changes in tPSA and f/t PSA Ratio Based on Use of Antibiotics

	tPSA (ng/mL)			
	n	Before	After	*P*
Antibiotics				
(+) Treatment	214	7.8 (1.7-123)	7.5 (2.9-90)	0.747
(-) Control	89	6.65 (3.32-73.64)	7.31 (3.52-91.09)	<0.001
*P*		0.081	0.401	
Antibiotics				
(+) Treatment	151	17 (3.6-47.6)	17 (3-37.5)	0.727
(-) Control	43	20.35 (6.56-31.2)	19.16 (7.39-54.6)	0.933
*p*		0.037	0.027	

**Table 2:** Distribution of Pathology Results in Treastment and Control Groups based on tPSA and f/t PSA Ratio Changes (based on cut-off value of 5%)

Antibiotics	tPSA	Pathology Result		*P*
		Prostate Cancer	Benign	
(+) Treatment	Decreased	24	59	0.407
	No change	13	22	0.498
	Increased	32	64	0.758
(-) Control	Decreased	7	9	0.614
	No change	3	12	0.112
	Increased	24	34	0.399
	f/tPSA Ratio			
(+) Treatment	Decreased	30	37	0.004
	No change	3	10	0.549
	Increased	16	55	0.014
(-) Control	Decreased	6	13	0.633
	No change	1	2	1.000
	Increased	5	16	0.558

**Table 3:** Distribution of Pathology Results in Treatment and Control Groups Based on f/t PSA Ratio Changes (Based on Cut-off Value of 10%)

Antibiotic	tPSA	Pathology result		*P*
		Prostate cancer	Benign	
(+) Treatment	Decreased	18	45	0.458
	No change	26	49	0.577
	Increased	25	51	0.880
(-) Control	Decreased	4	7	1.000
	No change	10	21	0.399
	Increased	20	27	0.372
	f/tPSA ratio			
(+)Treatment	Decreased	25	31	0.014
	No change	12	27	0.795
	Increased	12	44	0.026
(-) Control	Decreased	5	12	1.000
	No change	2	8	0.698
	Increased	5	11	0.737

**Table 4:** Evaluation of All Cases According to f/t PSA Ratio Changes (n=194)

	Pathology result		*P*
	Prostate cancer	Benign	
Changes on f/t PSA (cut off 5%)			
Decreased	36	50	0.005
Unchanged	4	12	0.562
Increased	21	71	0.014
Changes on f/t PSA (cut off 10%)			
Decreased	30	43	0.025
Unchanged	14	35	0.616
Increased	17	55	0.071

## Discussion

It has been reported that significant decreases in PSA levels can be seen following empirical use of antibiotics both in patients with chronic prostatitis and asymptomatic prostatitis (Schaeffer et al., 2005; Erol et al., 2006; Kaygısız et al., 2006; Serretta et al., 2008; Baltacı et al., 2009; Kyung et al., 2010; Stopiglia et al., 2010). In literature, there are some studies that significant decreases in PSA were observed following the antibiotherapy (Guercio et al., 2004; Kyung et al., 2010; Tang et al., 2010; Torky et al., 2011). In contrast, there are also studies reporting no significant PSA decreases with antibiotherapy as reported in this study (Bulbul et al., 2002; Dirim et al., 2009; Faydaci et al., 2012).

The greatest disadvantage of the studies investigating the effects of empirical antibiotherapy on high PSA levels, is the impossibility of understanding whether or not the PSA level decrease depending on the natural course of disease. In a study including a four-year follow-up reported that natural variations of PSA above or below 4 ng/mL were observed in 44% of the patients without treatment (Eastham et al., 2003). In the study of Komatsu et al., (1996) 814 cases were divided into 3 groups with PSA levels of 0-7.2 ng/m, 7.3-17.9 ng/m and >18.0 ng/m respectively, and physiological variations of 20%, 12% and 10%, respectively, were observed in the samples collected after a mean period of 80 days. In the current study, a significant result indicating an increase in PSA in the control group was reported. 

Beside the studies reporting that there are no significant differences between the control PSA levels of cases who had received antibiotic therapy and the control PSA levels left to the natural course as reported in this study (Shtricker et al., 2009; Stopiglia et al., 2010; Heldwein et al., 2011; Eggener et al., 2013; Toktas et al., 2013; Fandella et al., 2014; Yang et al., 2015; Greiman et al., 2016), there are also contradicting studies (Erol et al., 2006; Saribacak et al., 2014; Topac et al., 2016).

Varying conclusions have been reached in studies evaluating the f/t PSA ratio following antibiotherapy. As reported in current study, there are some studies that f/t PSA ratio did not display any significant changes following antibiotherapy (Serretta et al., 2008; Dirim et al., 2009; Faydaci et al., 2012). However, it has also been reported that this ratio has increased or decreased significantly after antibiotherapy (Lorente et al., 2002; Kaygısız et al., 2006; Kobayashi et al., 2008). 

There are limited number of studies in the literature observing the changes in f/t PSA ratio in patients who had not received antibiotics. In the studies of Busato et al. (2015) and Toktaş et al., (2013) no significant changes were found in the control f/t PSA ratio of patients not receiving antibiotherapy. The findings of this study are in concordance with the studies mentioned above.

Again varying results are noted in the limited number of studies evaluating the f/t PSA ratios in patients receiving and not receiving antibiotherapy. In the meta-analysis of Yang et al., (2015) and study of Heldwein et al., (2011), there are no significant differences in f/t PSA ratio between groups receiving and not receiving antibiotherapy. In contrast with this, another study have shown that significant f/t PSA ratio increases in the group receiving antibiotics (Lorente et al., 2002). Significant f/t PSA ratio decrease was observed in treatment group as compared to the control group in this study. 

The potential disadvantages of antibiotic administration include unnecessary expenses, side effects and possible adverse reactions related to the drug intake and an increase in multidrug resistant organisms (Scardino, 2007). Therefore, these conditions should be taken into consideration while administering antibiotics.

In a few studies that evaluating the histopathological results based on the PSA level changes obtained after antibiotherapy, it has been reported that there are no significant relations between the pathologic diagnoses and PSA levels as in this study for both cut off values (Dirim et al., 2009; Torky et al., 2011; Faydaci et al., 2012). On the other hand, it was found in the study of Serretta et al., (2008) that the rate of prostate cancer frequency in cases with PSA levels increasing or remaining unchanged following antibiotherapy was statistically higher as compared to those with decreasing PSA levels. In studies of Erol et al., (2006) and Sarıbacak et al., (2014) while the PSA decrease following antibiotherapy in cases diagnosed with BPH in the biopsy was statistically significant, no such significant difference was observed in cases diagnosed with cancer (Erol et al., 2006; Saribacak et al., 2014). 

In studies that pathology results were evaluated based on PSA changes in patients not receiving antibiotherapy, similarly to other studies in literature there were no significant relation between pathology results and PSA levels in this study for both cut off values (Shtricker et al., 2009; Eggener et al., 2013; Toktas et al., 2013; Saribacak et al., 2014).

If pathological results of treatment and control groups would be evaluated in terms of PSA changes; although Sarıbacak et al., (2014) reported that the PSA decrease in the group receiving antibiotics and with pathology results reported as BPH was found statistically significant as compared to the control group, similarly to many other studies in literature, there was no significant relationship between pathological results and PSA changes while taking the cut off values 5% and %10 in this study (Shtricker et al., 2009; Stopiglia et al., 2010; Heldwein et al., 2011; Eggener et al., 2013; Toktas et al., 2013; Fandella et al., 2014; Greiman et al., 2016).

There are some studies claiming that there is significant relation between f/t PSA ratio changes and histopathological results following antibiotherapy (Baltacı et al., 2009; Dirim et al., 2009), and also there are studies claiming that no such relation exists (Serretta et al., 2008; Faydaci et al., 2012; Toktas et al., 2013; Saribacak et al., 2014). In this study, when the cut-off value was taken as 5% for f/t PSA changes following antibiotherapy, prostate cancer rate was significantly higher in the group with decreasing ratios, while the benign results were significantly higher in the group with increasing ratios. A significant relation still existed when the cut-off value was taken as 10%. However, no significant relations were found between the f/t PSA ratio changes and histopathological results in the control group for both cut off values. This can be explained with the smaller number of cases in the control group. 

This study has some limitations. First of all, this study is retrospective, and the number of patients in the control group is small. Therefore, it is thought that significant results can be obtained from prospective studies involving larger patient groups. On the other hand, patients were not grouped using objective tools included the Chronic Prostatitis Symptom Index. Although the antibiotherapies administered to the patients were effective in urinary system infections, they were not the same in each case. Also, while the mean antibiotic use period was 4 to 6 weeks, they were not the same in each patient. Current systemic diseases of the patients included in the study and the other drugs they used were not recorded. Therefore, it is not known if the antibiotics have been affected by drug interactions.

In conclusion, serum PSA values are not correlated with histopathological results in patients receiving or not receiving antibiotics, and particularly, f/t PSA ratio changes in the group receiving antibiotics could be more useful in predicting the histopathological results. It is obvious that studies on larger series are required to evaluate the relation of PSA and ratio changes with the histopathological results when antibiotics are not administered. Although mpMRI is the first applied method according to guidelines in patients suspected with prostate cancer, these results may be a guide in places with limited accessibility to mpMRI, such as some developing countries.
